# Stimulation in the Dorsolateral Prefrontal Cortex Changes Subjective Evaluation of Percepts

**DOI:** 10.1371/journal.pone.0106943

**Published:** 2014-09-26

**Authors:** Tzu-Ching Chiang, Ru-Band Lu, Shulan Hsieh, Yun-Hsuan Chang, Yen-Kuang Yang

**Affiliations:** 1 The Brain and Mind Institute, The University of Western Ontario, London, Ontario, Canada; 2 Department of Psychology, National Chung Cheng University, Chiayi, Taiwan; 3 Department of Psychiatry, College of Medicine, National Cheng Kung University & Hospital, Tainan, Taiwan; 4 Institute of Behavioral Medicine, College of Medicine, National Cheng Kung University, Tainan, Taiwan; 5 Institute of Allied Health Sciences, College of Medicine, National Cheng Kung University, Tainan, Taiwan; 6 Department of Psychology, National Cheng Kung University, Tainan, Taiwan; The University of Queensland, Australia

## Abstract

Nelson and Narens have proposed a metacognition model that dissociates the objective processing of information (object-level) and the subjective evaluation of the performance (i.e., the metalevel). Neurophysiological evidence also indicates that the prefrontal cortices (PFC) are the brain areas which perform the metalevel function [1]–[3]. A corresponding neural mechanism of Nelson and Narens’s model, called dynamic filtering theory [4], [5], indicates that object-level processing is distributed in the posterior cortices and regulated by the prefrontal cortices with a filtering or gating mechanism to select appropriate signals and suppress inappropriate signals and noise. Based on this model, a hypothesis can be developed that, in the case of uncertainty or overloading of object-level processing, the prefrontal cortices will become more active in order to modulate signals and noise. This hypothesis is supported by a recent fMRI study [6] showing that the PFC (Brodmann area 9, BA9) was activated when subjects were overloaded in a bimodal attentional task, compared to a unimodal task. Here, we report a study showing that applying repetitive transmagnetic stimulation (rTMS) over the BA9 in order to interfere with its functional activity resulted in significant increas in guessed responses, compared to three other control conditions (i.e., no-TMS, sham TMS on BA9, and rTMS on Cz). The results are compatible with the dynamic filtering theory and suggest that a malfunction of the PFC would weaken the quality of meta-cognitive percepts and increase the number of guessed responses.

## Introduction

Perceptual confidence is a kind of metacognition in which subjects are aware of their performance, even errors, during response making [7]. Nelson and Narens [8], [9] proposed a metacognition model that dissociates the objective processing of information (object-level) and the subjective evaluation of performance (i.e., the metalevel). The function of the metalevel is to monitor, evaluate and initiate top-down controls to the object-level processing, in which the specific components of information processing, such as object recognition, spatial representation, and semantic processing, are executed. A corresponding neural mechanism of Nelson and Narens’s model, called dynamic filtering theory [4], [5], indicates that object-level processing is distributed in the posterior cortices and regulated by the prefrontal cortex (PFC) with a filtering or gating mechanism to select appropriate signals and suppress inappropriate signals and noises. The prefrontal cortices include “anterior PFC (BA10 or frontopolar PFC); dorsolateral PFC (BA9, BA46); ventrolateral PFC (BA44, BA45, BA47); dorsomedial PFC (BA24, BA32, or anterior cingulate cortex); and ventromedial PFC (BA11, BA12, or orbitofrontal cortex)” [10].

Neurophysiological evidence also indicates that the PFC is the brain area which performs the metalevel function ([1]–[3],see review [11]). When damage to the PFC occurs, subjective reports are decoupled from performance [12], [13]. As well, non-amnesic patients show poor metamemory accuracy (see review [2], [14]) and deficits in retrospective confidence judgements [15]. Recent studies further show negative correlations between rostrolateral PFC activity and confidence [16], [17] and that people in whom activity is higher in the lateral (rather than dorsomedial) PFC are likely to avoid cognitively demanding tasks [18].

Based on this model, a hypothesis can be developed that, given uncertainty or overloading of object-level processing, the prefrontal cortices will be more active in modulating signals and noise. This hypothesis is supported by a recent fMRI study [6] showing that the dorsolateral PFC (BA9) was more active when subjects performed bimodal tasks than in a combination of unimodal tasks. Here, we report a study showing that applying repetitive transcranial magnetic stimulation (rTMS) over the BA9 can reproduce the uncertainty effect while subjects perform a feature binding task. Although applying TMS on the dorsolateral PFC (DLPFC) has been done before by Rounis et al. [19], there were no anatomical control sites for the theta burst TMS. In earlier years, Turatto, Sandrini and Miniussi [20] used repetitive TMS (rTMS) on each side of the DLPFC and found that the stimulation of the right side of the DLPFC impaired detection of changed faces. This suggests that it may be involved in the process of visual awareness and working memory. Our hypothesis is that object-level processing in the parietal lobe is more efficient when the dorsolateral PFC is in a functional state, which also results in more confident responses in our feature binding task [6]. If in the meantime, we deliver rTMS over the dorsolateral PFC, compared to the other two control sites, it will disturb the recurrent feedback routes to the object processing and result in an increase in guessed responses.

## Methods

### Experimental setup and stimuli

Visual stimuli were adopted from Chiang et al. [6], consisting of 50 green and 50 red dots (or 50 yellow and 50 blue dots) on a black background, moving coherently at a speed of 4.3^o^/s in opposite directions along one axis. Equiluminance was separately established for each subject by flicker photometry [21]. Among the fifty dots of each colour, half moved randomly to increase the task difficulty. The stimuli in the rTMS experiment were presented on a PC with a 19-inch LCD monitor (800×600 pixels). All of the visual stimuli were constructed by COGENT Graphics (available at www.vislab.ucl.ac.uk) running in MATLAB (Mathworks Inc.).

The task required subjects to identify which color was moving and in what direction by using a right-hand keypad to answer one of two questions that randomly appeared after the stimulus presentation. One question asked, “Which color of dots was moving *direction*?” (The word *direction* was replaced by *up, down, left or right,* as appropriate.) The other question asked, “In which direction were the *color* dots moving?” (The word *color* was here replaced by *green, red, yellow* or *blue*, as appropriate.) Furthermore, the questions were relevant to the stimuli. For instance, if the subjects were shown red and green dots, they were not asked about the direction of blue or yellow dots, only that of red or green dots. If they were shown dots moving horizontally, they were not asked about the color of dots moving vertically. The combination of stimuli and related questions were balanced in a random sequence such that each type of stimulus was followed by each of the possible questions in turn. Meanwhile, subjects were also asked to report their level of confidence. A confident response was made by pressing the response key twice to increase the length of the response bar. A guessing response was made by pressing the response key once, resulting in a shorter bar. Subjects could freely change both their responses to the question and their reported level of confidence during the response period. Each subject’s response was categorized as either correct or incorrect based on its objectively assessed accuracy, and as either confident or guessing based on their subjective evaluation. Scores A, B, C and D in Table 1 represent the number of times the subjects’ responses fell into a particular category. For example, Score C would be the number of times that the subject made an incorrect response but stated that their response was confident, and Score B the number of times that a subject responded correctly, but reported that the response was a guess.

**Table 1:** Response categories.

		Subjective Evaluation	
		Confident	Guessing
**Objective Evaluation**	Correct	A	B
	Incorrect	C	D

Responses were categorized according to the subjects’ assessment of their confidence in their ability to bind features and correctness in binding. Confident responses were represented as the combination of Cell A and Cell C, while guessing responses were represented as the combination of Cell B and Cell D.

### Subjects

Five healthy subjects (3 males and 2 females) between 19–38 years of age (mean 25.0, SD 7.7) participated in the rTMS study. All subjects were right-handed and had normal or corrected to normal vision. All gave written informed consent in accordance with the Declaration of Helsinki and ethical consent for the rTMS study was granted by the Human Experiment and Ethics Committee of National Cheng Kung University Hospital (IRB number: ER-98-162).

### TMS equipment and structure imaging details

The TMS stimulator was a Magstim Rapid^2^ (Whitland, Dyfed, UK). Magnetic stimulation was applied at 60% of the maximum output for 5 pulses in 500 ms using a double figure-of-eight 70-mm cooled coil. A double placebo 70-mm coil was adopted for the sham stimulation.

**Figure 1:**
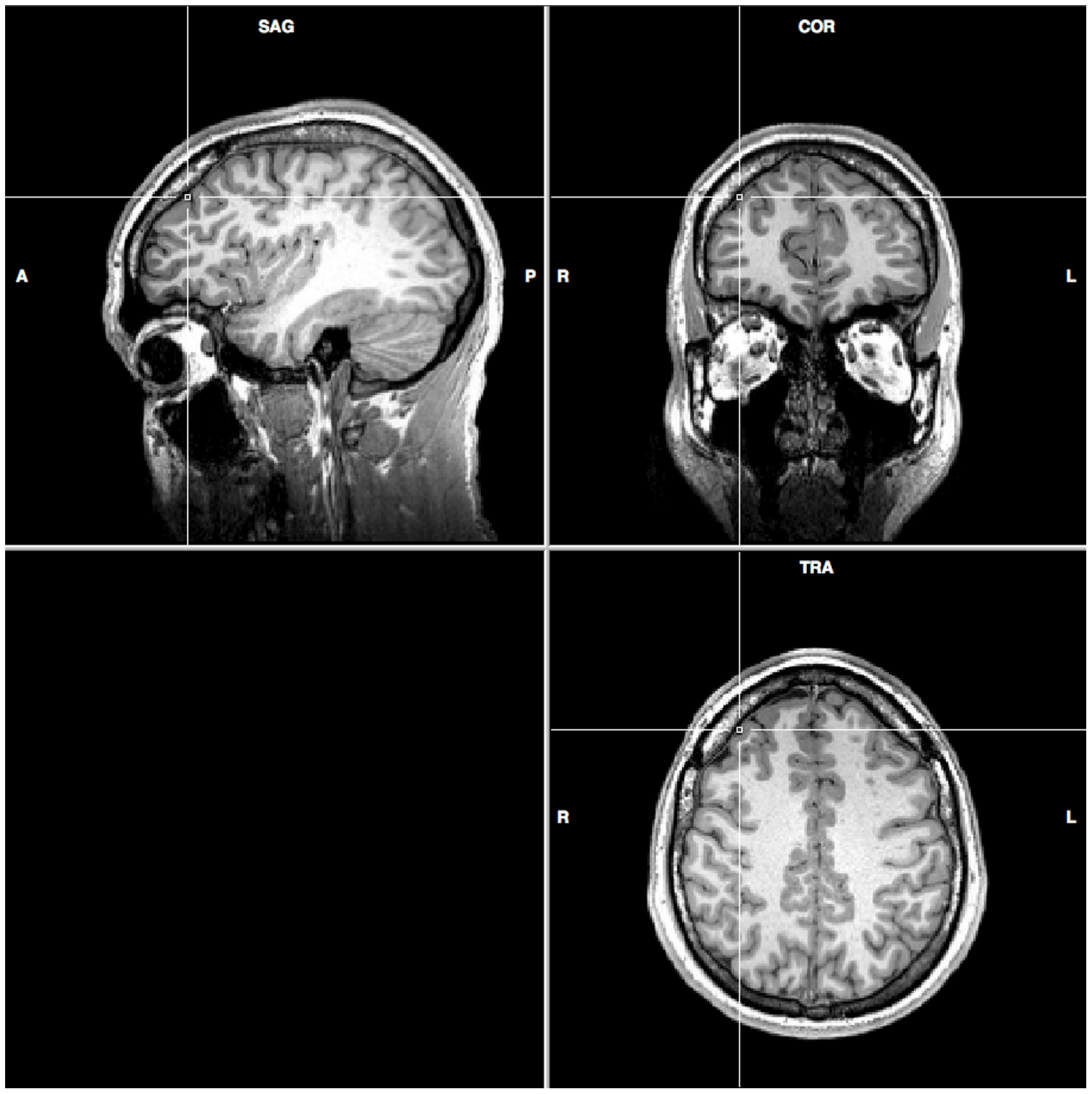
Targeting the dorsolateral PFC, depicted at the cross, for rTMS with a volume view from a subject’s anatomical brain images. The centre of the rTMS coil is vertically attached to the skull, in order to get the shortest distance to the target. The coordinates of the target are (37, 39, 40) in Talairach space.

Before TMS stimulation, each subject received structure scans in the 3T Bruker 30/90 Medspec fMRI scanner fitted with a standard birdcage head coil (BrukerBioSpin MRI GmbH, Ettlingen, Germany). The structure images were a T1 weighted axial anatomical scanning (resolution = 0.9375×0.9375×3.75 mm, TE = 39.4 ms, TR = 614.2 ms, flip angle 90 degrees, FOV = 240 mm). The anatomical scans were used to map the TMS coil to the dorsolateral PFC (Figure 1) by using the Brainsight system (Rogue Resolutions Ltd, Cardiff, Wales, UK) and the Polaris Optical Tracking system (Northern Digital, Inc., Waterloo, Ontario, Canada).

### Procedure and data analysis

Before starting the rTMS stimulation, we determined an optimal stimulus duration for the feature binding task which allowed the subject to identify the stimulus correctly in 70% of the presentations that were declared as confident. We used transformed up-down procedures [22] to vary the stimulus duration. When the subjects’ response was incorrect the duration of the next stimulus was increased and, conversely, the duration was decreased after two successive correct responses. With this approach, the stimulus duration among subjects varied among subjects (from 50 ms to 150 ms, mean 90 ms, SD 41.8 ms).

The rTMS experiment was conducted on a PC in a dark room. Subjects sat with their head supported by a chinrest. Prior to the rTMS study, the scalp was marked to identify the location of the right dorsolateral cortex (BA9) according to the BrainSight software and the individual structure scans. There were 4 blocks of 32 trials with the following randomized conditions: no-TMS, rTMS over BA9, rTMS on Cz (the top of head using the 10–20 system), and sham TMS over the BA9.

Each trial started with a cross presented for 500 ms. Then the visual stimuli were presented for a chosen duration. The visual stimuli subsequently disappeared and a question appeared at the bottom of the screen for a 6-second period, during which the subjects responded to the question and gave confidence ratings with a right hand keypad. The next trial automatically began after the 6 seconds had passed.

### Data analysis

The non-linear mixed effect model (NLMX) was adopted because it was more appropriate than the more traditional general linear model (GLM) or analysis of variance (ANOVA) [23], [24]. The distribution of subjects’ responses was modeled by an equation consisting of one variable (the stimulation site) with 4 conditions: no-TMS, rTMS on BA9, sham TMS on BA9, and rTMS on Cz.
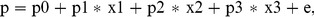
(1)where *p* was the ratio of the number of trials with guessed responses to the total number of trials per stimulation site. *x1*, *x2* and *x3* were the coding of stimulation sites, [0 0 0] represented the no-TMS condition, [1,0,0] was equivalent to the rTMS stimulation on BA9, [0,1,0] was the representative of the sham TMS over BA9, and finally [0,0,1] was the coding of the rTMS over Cz stimulation. *e* was the variance among subjects and was assumed to fit the standard normalised distribution. Coefficient *p0* was the estimated probability of responses in the no-TMS condition. Similarly, the coefficients *p1, p2* and *p3* were the estimated probability deviations from *p0* for the BA9, sham and Cz stimulation, respectively. The NLMX model was analyzed with SAS software (SAS Institute Inc. Cary, NC, USA).

## Results

**Figure 2:**
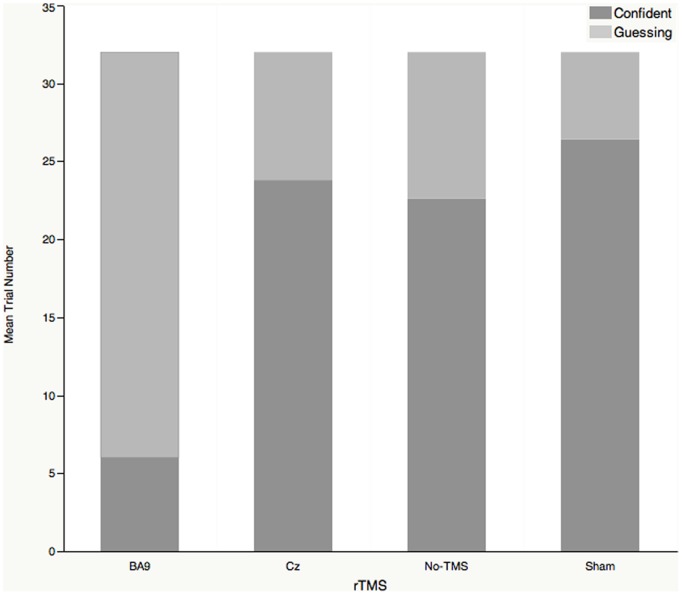
The number of confident and guessed responses in the rTMS experiment, grouped by stimulation site. The total number of trials for each subject was 32. The number of guessed trials significantly increased only while rTMS was over BA9 and not for the sham TMS or Cz stimulation, compared with the no-TMS condition (the non-linear mixed effect model was adopted, t_4_ = 11.31, p = 0.0003). Cz is the top of the head, according to the 10–20 EEG system.

The distribution of trial numbers in the four TMS conditions is displayed in Figure 2. In the no-TMS condition, most of the subjects’ responses were confident (a mean of 22.6 out of 32 trials, SD 5.32), while the guessed responses were an average of 9.4 out of 32 trials. Applying rTMS over the right dorsolateral PFC resulted in a significant increase of guessed responses over confident responses (a mean of 26 guessing responses and 6 confident responses) (t_4_ = 11.31, p = 0.0003). In contrast, delivering a sham TMS coil over the dorsolateral PFC or rTMS over Cz did not change the distribution of guessed and confident responses, compared to the no-TMS condition (t_4_ = –.1.64 & –0.52, p = 0.18 & 0.63, respectively). In a word, the number of guessed responses significantly increased only for rTMS over the dorsolateral PFC and not for the sham TMS and Cz stimulation.

**Figure 3:**
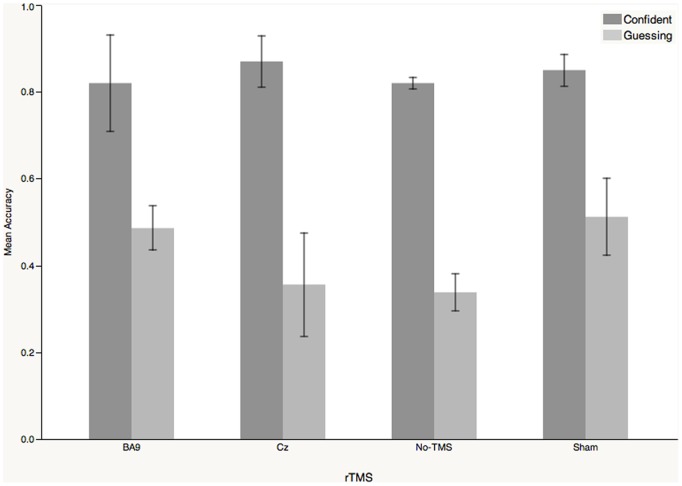
The distribution of accuracy in the rTMS experiment, grouped both by the factor of confident or guessed responses and by stimulation site. The error bar of each column indicates the standard error of the data. The accuracies of the guessed trials among the four stimulation sites were similar, all at the chance level (the non-linear mixed effect model was adopted, rTMS on BA9 versus no-TMS, t_4_ = 1.64, p = 0.1768; sham TMS on BA9 versus no-TMS, t_4_ = 0.57, p = 0.5980; rTMS on Cz versus no-TMS, t_4_ = 0.67, p = 0.5378).

The accuracy data is displayed in Figure 3. In the no-TMS condition, the mean accuracy was 0.82 (SD = 0.031) for the confident responses and 0.34 (SD = 0.097) for the guessed responses. The accuracy pattern of the other three conditions stayed the same as the no-TMS condition (in the guessed responses: rTMS on the BA9 versus no-TMS, t_4_ = 1.64, p = 0.1768; sham TMS on the BA9 versus no-TMS, t_4_ = 0.57, p = 0.5980; rTMS on Cz versus No-TMS, t_4_ = 0.67, p = 0.5378; in the confident responses: rTMS on the BA9 versus no-TMS, t_4_ = 0.015, p = 0.9888; sham TMS on the BA9 versus no-TMS, t_4_ = 0.83, p = 0.4532; rTMS on Cz versus no-TMS, t_4_ = 0.88, p = 0.4286). In a word, the accuracy of both confident and guessed responses did not change, no matter where the rTMS coil was placed.

## Discussion

The study aimed to verify the modulation effects of the dorsolateral PFC on object level processing postulated by Shimamura [4], [5]. There were 2 results. One was that rTMS on the dorsolateral PFC changed the distribution of confidence and guessing categories, by increasing the number of guessed responses compared to the other three control conditions (i.e., no-TMS, sham TMS, and rTMS on Cz). It suggests that a malfunction of the dorsolateral PFC would cause wrong judgments about current object-level processing and result in wrong subjective evaluation/awareness of perception. The other result from our study was that the accuracy of confident and guessed responses remained the same as in the other control conditions, even the trial number of the confident and guessing responses reversed under rTMS. This indicates that the function of the dorsolateral PFC is not to operate features in object-level processing. If it were, the accuracy of the responses under rTMS would have changed, rather than staying at the same level as the other control conditions. The results also back up the dynamic filtering theory and clearly indicate the metalevel function of the dorsolateral PFC, which is independent from object-level processing.

Our data analysis did not adopt the type 2 signal detection theory (SDT) because the current popular models about type 2 SDT are developed from type 1 SDT, which requires two different physical stimuli, for example ‘signal+noise’ and ‘noise’ (or S1 and S2) [12], [25]. The current study has only one identical stimulus. In this situation, the ‘miss’ and ‘correct rejection’ (CR) in SDT (type 1 SDT) did not apply to the ‘guessing’ condition in our study. In a classic book, *Detection Theory: A User’s Guide, 2nd Ed.* [26], the type 2 SDT was introduced (pp. 73–74), “… but because there is only one stimulus class (the words on the original list), no type-1 curve is possible.” (p.74). One effective way to examine guessed responses is their accuracy. If the accuracy of the guessed responses is around chance level, the declaration of guessing is trustworthy. It also suggests that the participants have no conscious knowledge of the binding [27]. Nevertheless, if the accuracy of the guessed responses is either significantly below or above the chance level, there will be either a response bias in subjects’ decision making or a blindsight phenomenon. The blindsight phenomenon in normal subjects is still a controversial issue [28], [29].

The results of rTMS over the BA9 not only back up the results from patients’ data showing that the dorsolateral PFC is the site of meta-cognition, but also offer better physiological evidence in Nelson’s model [8], [9] with more appropriate control sites. In addition, unlike Rounis’s ([19]) placing TMS in between blocks of visual presentation, our experiment applies TMS at the time when subjects are viewing visual stimuli. Realtime TMS over DLPFC has been executed before in a face change detection task [20] in which, under the application of rTMS, the percentage of correct detection trials was reduced but the detection of change-absent trials still remained a high percentage of trial numbers. Our results are in line with Turatto et al.‘s (2004) in terms of the distribution of confident and guessed responses within the trials. However, our results further provide the unchanged accuracy of both confident and guessed responses, which can avoid the confounding variable, response bias, when subjects make a decision on each trial. Otherwise, the change in distribution of the responses would be confounded by a response bias due to the conservative outcomes of attention [30], i.e., that subjects are likely to make guessed responses during the stimulation of rTMS, rather than experiencing a change of perceptual awareness of stimuli. Other supporting evidence comes from a recent paper [31] that applied 1 Hz TMS over DLPFC to reduce its activity, resulting in an increased acceptance of hypnotic suggestion; however, the TMS effect did not change subjects’ expectancy about their own suggestibility when asked about it before the hypnotic induction.

In a word, our results reveal that rTMS causes the dorsolateral prefrontal cortex to wrongly evaluate object processing, resulting in an increased number of guessed responses. This implies that the dorsolateral prefrontal cortex may be the gatekeeper in modulating the final judgement of object processing. Further research will be necessary to explore the interaction of metalevel and object-level processing in terms of brain function.

## Supporting Information

Data S1 Raw Data.
(XLSX)
